# Disease-Associated Gut Microbiota Reduces the Profile of Secondary Bile Acids in Pediatric Nonalcoholic Fatty Liver Disease

**DOI:** 10.3389/fcimb.2021.698852

**Published:** 2021-09-09

**Authors:** Jiake Yu, Hu Zhang, Liya Chen, Yufei Ruan, Yiping Chen, Qi Liu

**Affiliations:** Department of Pediatric Infectious Disease, The Second Affiliated Hospital & Yuying Children’s Hospital of Wenzhou Medical University, Wenzhou, China

**Keywords:** pediatric nonalcoholic fatty liver disease, gut microbiota, metagenomics sequencing, bile acid metabolism, bile salt hydrolase, 7α-dehydroxylase

## Abstract

Children with nonalcoholic fatty liver disease (NAFLD) display an altered gut microbiota compared with healthy children. However, little is known about the fecal bile acid profiles and their association with gut microbiota dysbiosis in pediatric NAFLD. A total of 68 children were enrolled in this study, including 32 NAFLD patients and 36 healthy children. Fecal samples were collected and analyzed by metagenomic sequencing to determine the changes in the gut microbiota of children with NAFLD, and an ultra-performance liquid chromatography coupled to tandem mass spectrometry (UPLC-MS/MS) system was used to quantify the concentrations of primary and secondary bile acids. The associations between the gut microbiota and concentrations of primary and secondary bile acids in the fecal samples were then analyzed. We found that children with NAFLD exhibited reduced levels of secondary bile acids and alterations in bile acid biotransforming-related bacteria in the feces. Notably, the decrease in Eubacterium and Ruminococcaceae bacteria, which express bile salt hydrolase and 7α-dehydroxylase, was significantly positively correlated with the level of fecal lithocholic acid (LCA). However, the level of fecal LCA was negatively associated with the abundance of the potential pathogen *Escherichia coli* that was enriched in children with NAFLD. Pediatric NAFLD is characterized by an altered profile of gut microbiota and fecal bile acids. This study demonstrates that the disease-associated gut microbiota is linked with decreased concentrations of secondary bile acids in the feces. The disease-associated gut microbiota likely inhibits the conversion of primary to secondary bile acids.

## Introduction

Nonalcoholic fatty liver disease (NAFLD) is the liver manifestation of obesity-related metabolic diseases. NAFLD is characterized by a broad spectrum of disorders, ranging from simple steatosis to nonalcoholic steatohepatitis (NASH) with inflammation and fibrosis, which may progress to hepatic cirrhosis, and ultimately liver failure (Brunt, 2010). NAFLD is one of the most common chronic liver diseases in children worldwide and affects about 3-10% of children (Nobili et al., 2016). Pediatric studies have suggested that an unhealthy lifestyle is likely to be the main trigger of NAFLD in children, which includes the high consumption of carbohydrates and/or high-fat and fried foods combined with sedentary behavior (Mollard et al., 2014). Animal models and genome-wide association studies have expanded our understanding of NAFLD, but the pathogenesis of this disease remains obscure (Arab et al., 2018).

Multiple lines of evidence have shown that the gut microbiota community plays a crucial role in NAFLD pathophysiology (Bluemel et al., 2016; Jiao et al., 2018). A recent metagenomic sequencing study revealed altered gut microbial composition and functional annotations in children with NAFLD at the fecal level, which were significantly different from that found in healthy children (Zhao et al., 2019). Moreover, a recent 16S rRNA sequencing study showed that NAFLD was associated with altered gut microbiota and concentrations of serum bile acids, which contributed to impaired hepatic bile acid-mediated signaling in NAFLD livers (Jiao et al., 2018). Bile acids are now known not only to facilitate the digestion and absorption of fat-soluble vitamins and dietary fats in the intestine, but also serve as important signaling molecules that regulate NAFLD pathophysiology (Arab et al., 2017). However, evidence of a fecal bile acid signature in NAFLD patients is lacking (Arab et al., 2018). In humans, the gut microbiota deconjugate bile acids and convert primary bile acids, cholic acid (CA), and chenodeoxycholic acid (CDCA) into secondary bile acids, such as deoxycholic acid (DCA) and lithocholic acid (LCA), and therefore it has a potent effect on bile acid signaling (Ridlon et al., 2006). Additionally, gut microbiota dysbiosis has been shown to exert a profound effect on the bile acid profile in several gut microbiota-related diseases (Wang et al., 2019; Liwinski et al., 2020). Nevertheless, to the best of our knowledge, the relationship between gut microbiota dysbiosis and bile acid composition in pediatric NAFLD remains to be elucidated.

In this study, we aimed to examine the composition of the gut microbiota in selected cohorts of children with NAFLD and healthy controls. Furthermore, we assayed the fecal bile acid profiles in these two groups to analyze potential associations between the gut microbiota and bile acid metabolism in pediatric NAFLD.

## Materials and Methods

### Study Subjects

A total of 68 children from the Second Affiliated Hospital and Yuying Children’s Hospital of Wenzhou Medical University, Wenzhou, China, were recruited for the study from October 2018 to June 2020 (**Table 1**). The diagnosis of NAFLD in children was based on ultrasound findings and elevated transaminase levels, as previously described (Malespin et al., 2015; Michail et al., 2015; Nobili et al., 2019). An abnormal transaminase level was defined as alanine aminotransferase (ALT) levels >25.8 IU/L for boys and >22.1 IU/L for girls (Malespin et al., 2015). Thirty-six matched healthy children without any history of chronic liver disease were enrolled in the study as controls. All subjects who took antibiotics or probiotics within 3 months prior to fecal collection were excluded. Exclusion criteria also included those with current use of immunosuppressants, major gastrointestinal tract surgery, active gastrointestinal and liver disease, and/or diabetes. This study was carried out in accordance with the ethical guidelines of the Declaration of Helsinki and was approved by the Ethical Review Boards of the Second Affiliated Hospital and Yuying Children’s Hospital of Wenzhou Medical University. All parents and legal guardians of the participants provided written informed consent before sample collection.

**Table 1:** Subject characteristics.

Characteristics	HC (n=36)	NG (n=32)	P value
**Demographics**			
**Age, years, median (range)**	12 (5−16)	11 (8−15)	0.337
**Sex, male, n (%)**	21 (58.33%)	20 (62.50%)	0.726
**BMI, kg/m2, median (range)**	17.88 (14.24−28.11)	25.62 (19.15−45.92)	**0.047**
**Hypertension (%)**	0	42.86	**0.004**
**Blood lipids, median (range)**			
**Triglycerides, mmol/L**	0.74 (0.39−2.37)	1.46 (0.33−3.49)	**0.002**
**Total cholesterol, mmol/L**	3.72 (2.69−6.04)	4.94 (3.21−8.98)	0.160
**High density lipoprotein (HDL), mmol/L**	1.31 (0.82−2.2)	1.16 (0.64−2.81)	0.882
**Low density lipoprotein (LDL), mmol/L**	1.95 (0.98−4.26)	2.91 (1.46−6.83)	0.241
**Hepatic function, median (range)**			
**ALT, U/L**	11 (6−53)	53 (23−393)	**0.001**
**AST, U/L**	21 (11−53)	36 (21−185)	**0.001**
**AKP, U/L**	226 (49−446)	274 (75−429)	0.134
**GGT, U/L**	11 (7−38)	37 (15−271)	**0.002**
**TB, µmol/L**	10.8 (4.7−31.5)	10.3 (4.1−22.2)	0.091
**ALB, g/L**	46.6 (43.7−49.9)	47.5 (39.2−51.9)	0.061
**TBA, µmol/L**	3.0 (1.8−7.6)	3.84 (1.4−9.3)	0.063
**Glucose, mmol/L**	4.74 (4.21-5.82)	4.98 (3.92−6.51)	**0.002**
**Insulin, µIU/mL**	6.71 (2.83-23.7)	32.25 (8.88−202)	**0.004**

ALT, alanine aminotransferase; AST, aspartate aminotransferase; AKP, alkaline phosphatase; GGT, gamma-glutamyltransferase; TB, total bilirubin; ALB, albumin; TBA, total bile acid; HC, health controls; NG, NAFLD group. A bold P value was considered significant.

### Metagenomics Sequencing and Analysis of Fecal Microbiome

Fecal samples were collected with a sterile kit and immediately stored at −80°C. Microbial DNA was extracted from 68 fecal samples using the E.Z.N.A.^®^ Stool DNA Kit (Omega Bio-tek, Norcross, GA, USA) according to the manufacturer’s protocol. Metagenomic shotgun sequencing libraries were constructed and sequenced at the Shanghai Biozeron Biological Technology Co. Ltd. Briefly, for each sample, 1 μg of genomic DNA was sheared using a Covaris S220 Focused-Ultrasonicator (Woburn, MA, USA), and sequencing libraries were prepared with a fragment length of approximately 450 bp. All samples were sequenced using the Illumina HiSeq 2500 instrument with pair-end 150 bp (PE150) mode. Raw sequence reads underwent quality trimming using Trimmomatic (http://www.usadellab.org/cms/uploads/supplementary/Trimmomatic) to remove adaptor contaminants and low-quality reads. Reads that aligned to the host genome were also removed. The remaining reads were called clean reads and were used for further analysis. An average of 9.52 gigabase (Gb) paired-end reads were obtained for each sample, totaling 371.2 Gb of high-quality data that were free of host DNA and adaptor contaminants.

The overall distinctions in microbial composition were measured through PCoA of a Bray-Curtis distance. LEfSe was applied to compare significant differences in taxa between NAFLD patients and healthy subjects, using a P value <0.05 and an LDA score >3 (Segata et al., 2011). BLAST (BLAST Version 2.2.28+) was used to link the metagenomics sequencing datasets and KEGG databases. We then used KOBAS 2.0 (KEGG Orthology Based Annotation System 2.0) to predict the bile-metabolizing function of gut microbiota within the samples (Xie et al., 2011). Predicted gene abundance was evaluated for KEGG orthology K01442 (BSH), KEGG orthology K15870 (baiCD), and KEGG orthology ko00121 (involving the secondary bile acid biosynthesis pathway). The Mann-Whitney U test was applied to evaluate the differences in the relative abundances of taxa and the level of bile acids between the two groups for statistical significance. The relationships between the structure of the intestinal microbiota and fecal bile acids were assessed by Spearman’s correlation analysis.

### Fecal Bile Acid Analysis

Targeted metabolomics profiling was performed to measure the concentrations of 42 bile acids in fecal samples based on previously described methods (Xie et al., 2013). Briefly, fecal samples (10 mg) were homogenized with 200 μL acetonitrile/methanol (8:2 v/v) mixture, containing 10 μL internal standard, using a Bullet Blender Tissue Homogenizer (Next Advance, Inc., Averill Park, NY, USA), and centrifuged at 13,500 rpm (4°C) for 20 min. After centrifugation, 10 μL of each supernatant was diluted with 90 μL of 1:1 (v/v) mobile phase mixture (where phase A=acetonitrile/methanol/isopropanol (8:1:1 v/v/v); and B=ammonium acetate with 0.25% acetate acid). After centrifugation, 5 μL supernatant was transferred to a 96-well plate for analysis with a UPLC-MS/MS system (ACQUITY UPLC-Xevo TQ-S, Waters Corp., Milford, MA, USA), which was used to quantitate bile acids. Data processing was performed using MassLynx software (v4.1, Waters, Milford, MA, USA). The ACQUITY UPLC Cortecs C18 column (1.6 μm, 100 mm × 2.1 mm internal dimensions) (Waters, Milford, MA) was used to achieve chromatographic separation. The UPLC-MS raw data acquired in negative mode were processed using TargetLynx application manager (Waters, Milford, MA) to obtain calibration equations and accurate concentrations of each bile acid in the fecal samples.

### Statistical Analysis

All statistical analyses were conducted using SPSS software v.25. The data are expressed as median (range) with standard deviations (± SD) and compared using the t test (for normally distributed data) or the Wilcoxon test (for not normally distributed data). P values <0.05 were considered statistically significant.

## Results

### Clinical Characteristics of Children With NAFLD

A total of 68 children were enrolled in the study, 41 (60.3%) of which were boys (**Table 1**). The median age at diagnosis was 11 years (range, 8-15 years). We divided all subjects into two groups, including patients with NAFLD (NG, n=32) and healthy controls (HC, n=36). There was no significant difference in sex and age between the two groups. Children with NAFLD had a higher body mass index (BMI) than healthy children. Blood tests showed higher levels of alanine aminotransferase (ALT; P<0.001), aspartate aminotransferase (AST; P<0.001), and gamma-glutamyltransferase (GGT; P<0.001) compared to healthy children. Elevated levels of triglycerides (TG; P<0.01), glucose (P<0.01), and insulin (P<0.01) were also observed in children with NAFLD. Additionally, children with NAFLD had a higher proportion of hypertension compared to healthy children (**Table 1**).

### Gut Microbiota Dysbiosis in Children With NAFLD

**Figure 1:**
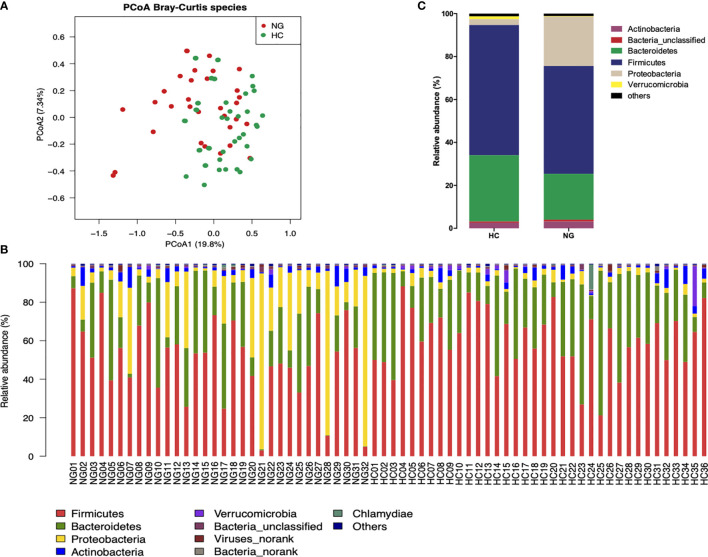
Differences in the fecal microbiota composition of children with NAFLD (NG) and heathy control (HC) children. **(A)** Principal-coordinate analysis (PCoA) of a Bray-Curtis distance generated from fecal bacterial taxa shown at the species level. **(B)** Phyla distribution of gut microbiota in the NAFLD (n=32) and HC (n=36) children. The distribution of gut bacterial phyla (abundance >1%) of each sample is shown as a bar chart in terms of percentage weight. **(C)** Average relative abundance of gut microbiota at the phylum level in NG (n=32) and HC (n=36) children.

**Figure 2:**
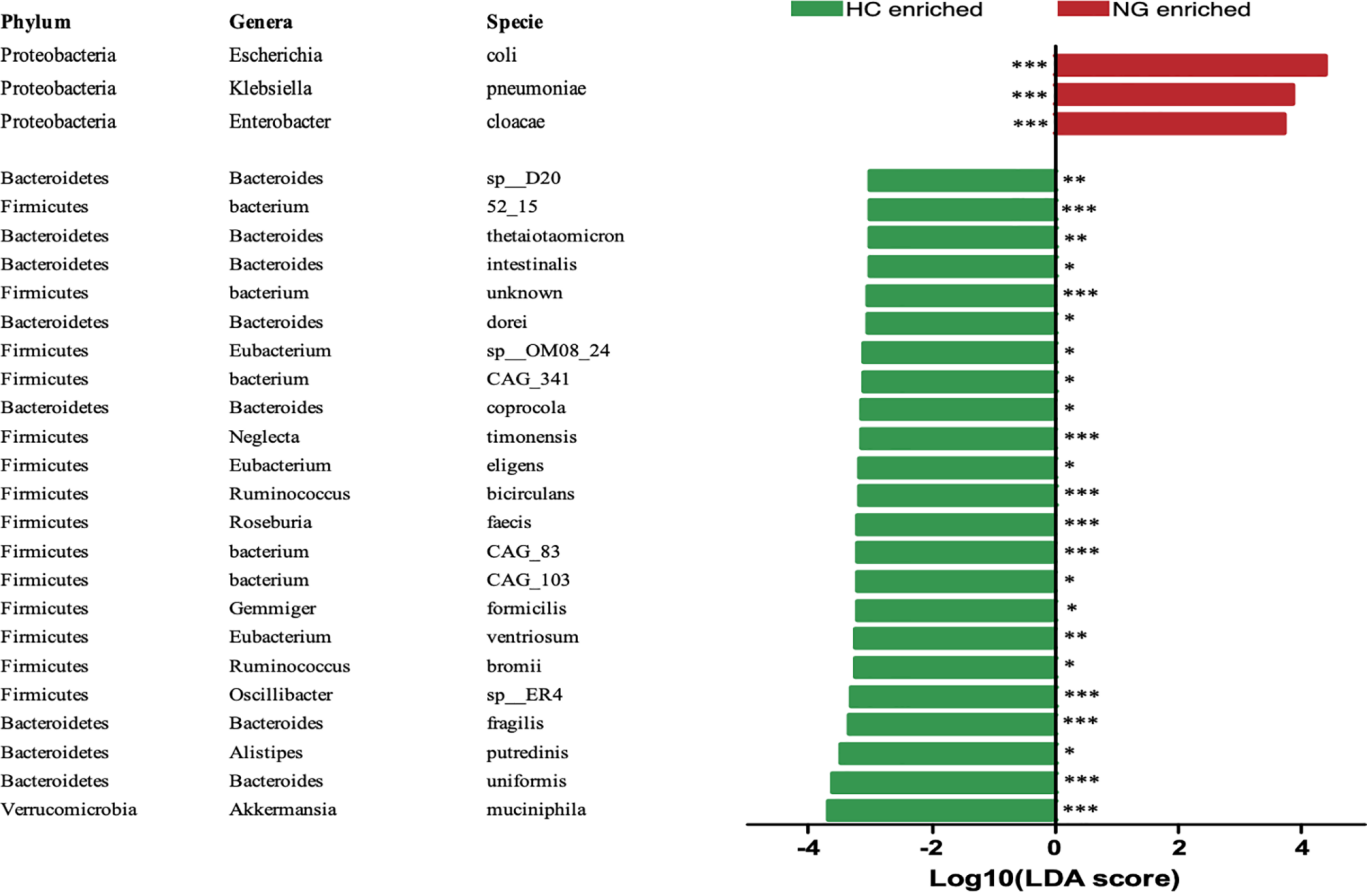
LEfSe analysis showed that the relative abundances of 26 species were significantly distinct between the two groups. *P < 0.05; **P < 0.01; ***P < 0.001. The Wilcoxon test was performed for comparisons between the two groups. HC, healthy controls; NG, NAFLD group; LEfSe, linear discriminant analysis (LDA) effect size method (LEfSe).

To assess the changes in the gut microbiota, we performed metagenomic sequencing of 68 fecal samples from children with NAFLD (n=32) and healthy control children (n=36). Principal coordinate analysis (PCoA) of a Bray-Curtis distance generated from species-level taxa showed a significant separation in gut microbial composition between children with NAFLD and HC children (P<0.05, **Figure 1A**). At the phylum level, we found that Firmicutes, Bacteroidetes, Proteobacteria, and Actinobacteria were the dominant phyla in children with NAFLD and HC (**Figure 1B**). We observed significant changes at the phylum level between the two groups. The relative abundance of Firmicutes (P<0.05) and Bacteroidetes (P<0.05) was reduced and Proteobacteria (P<0.001) was increased in children with NAFLD (**Figure 1C**). At the species-level, we performed linear discriminant analysis (LDA) effect size method (LEfSe) analysis on the fecal bacterial composition and obtained 26 bacterial taxa with significantly different relative abundances between the two groups (LDA score >3.0, P<0.05) (**Figure 2**). Children with NAFLD showed increased abundance of three bacterial taxa, including *Escherichia coli* (LDA score=4.37, P<0.001), *Klebsiella pneumoniae* (LDA score=3.84, P<0.001), and *Enterobacter cloacae* (LDA score=3.71, P<0.001), compared with the HC children. The levels of some potentially beneficial bacteria, such as *Akkermansia muciniphila* and *Alistipes putredinis*, were significantly reduced in children with NAFLD. In addition, the relative abundances of species *Bacteroides uniformis*, *Bacteroides fragilis*, *Oscillibacter* sp. *ER4*, *Ruminococcus bromii*, *Eubacterium ventriosum*, and *Gemmiger formicilis* were also significantly decreased in children with NAFLD compared with the HC group.

### Disease-Associated Gut Microbiota Is Correlated With Low Abundances of Bile Acid-Biotransforming-Related Gene

**Figure 3:**
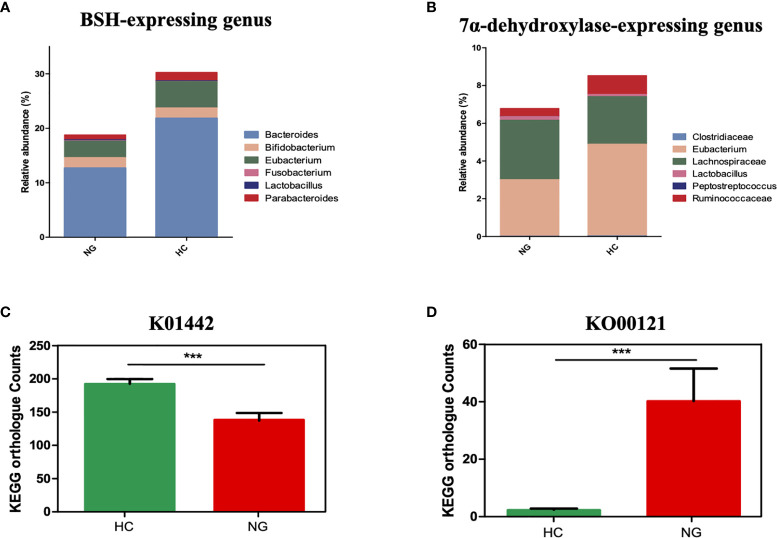
The association between a Eubacterium-depleted microbiota and bile acid biotransformation in children with NAFLD. **(A)** The relative abundances of bile acid hydrolase (BSH)-active gut bacteria. **(B)** The relative abundances of 7α-dehydroxylating gut bacteria. **(C)** Inference function of BSH KEGG ortholog counts (K01442) (p < 0.001). **(D)** Inference function of secondary bile acid biosynthesis KEGG ortholog counts (ko00121) (P < 0.001). ***P < 0.001.

KOBAS 2.0 (Kyoto Encyclopedia of Genes and Genomes (KEGG) Orthology Based Annotation System 2.0) was applied to predict the gene abundance of KEGG functions in bile acid metabolism. We found that the bile salt hydrolase (BSH) gene (KEGG orthology K01442, deconjugating bile acids) was significantly lower in children with NAFLD than that in HC (**Figure 3C**), indicating a decrease of bacterial population that harbors BSH enzymes. We analyzed BSH-expressing genus levels of bacteria and found that they were markedly lower in NAFLD children than in HC. Specifically, we found that the major BSH-expressing bacterial genera, *Bacteroides *(P<0.01) and *Eubacterium *(P<0.05), were significantly decreased in NAFLD children (**Figure 3A**).

7α-dehydroxylase is a major enzyme that catalyzes the generation of secondary bile acids, such as DCA and LCA, from primary bile acids by 7α-dehydroxylation. Although the baiCD gene (KEGG orthology K15870), known to encode 7α-dehydroxylase, was not accurately predicted for all samples, likely due to the low abundance, the genus levels of bacteria that harbor 7α-dehydroxylase gene were decreased in NAFLD children compared to HC. Specifically, *Eubacterium*(P<0.05) and *Ruminococcaceae* (P<0.05) were lower in NAFLD group (**Figure 3B**). Interestingly the predicted gene abundance for secondary bile acid biosynthesis (KEGG orthology ko00121) was significantly higher in children with NAFLD than in HC children (**Figure 3D**, P<0.001, Mann-Whitney U test). Taken together, these results showed that children with NAFLD had significant lower *Bacteroides*, *Eubacterium*, and *Ruminococcaceae*, and decreased bile acid deconjugation and dehydroxylation activity, indicating that NAFLD children may have elevated conjugated bile acids and lowered secondary bile acids in their feces.

### Lower Levels of Fecal Secondary Bile Acids in Pediatric NAFLD With Reduced Ratio of LCA/CDCA

**Figure 4:**
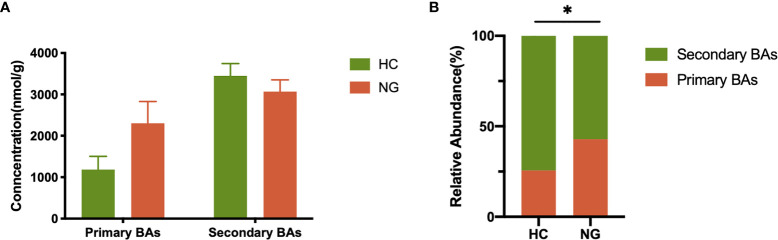
Alteration of fecal primary and secondary bile acid profiles in NAFLD children. **(A)** Profiles of fecal primary and secondary bile acids in pediatric NAFLD patients (n=32) and healthy controls (n=36). **(B)** Proportions of fecal primary and secondary bile acids. The relative abundance of fecal bile acids was evaluated between the two groups by the Mann-Whitney test. *P < 0.05. HC, healthy controls; NG, NAFLD group; BAs, bile acids.

**Figure 5:**
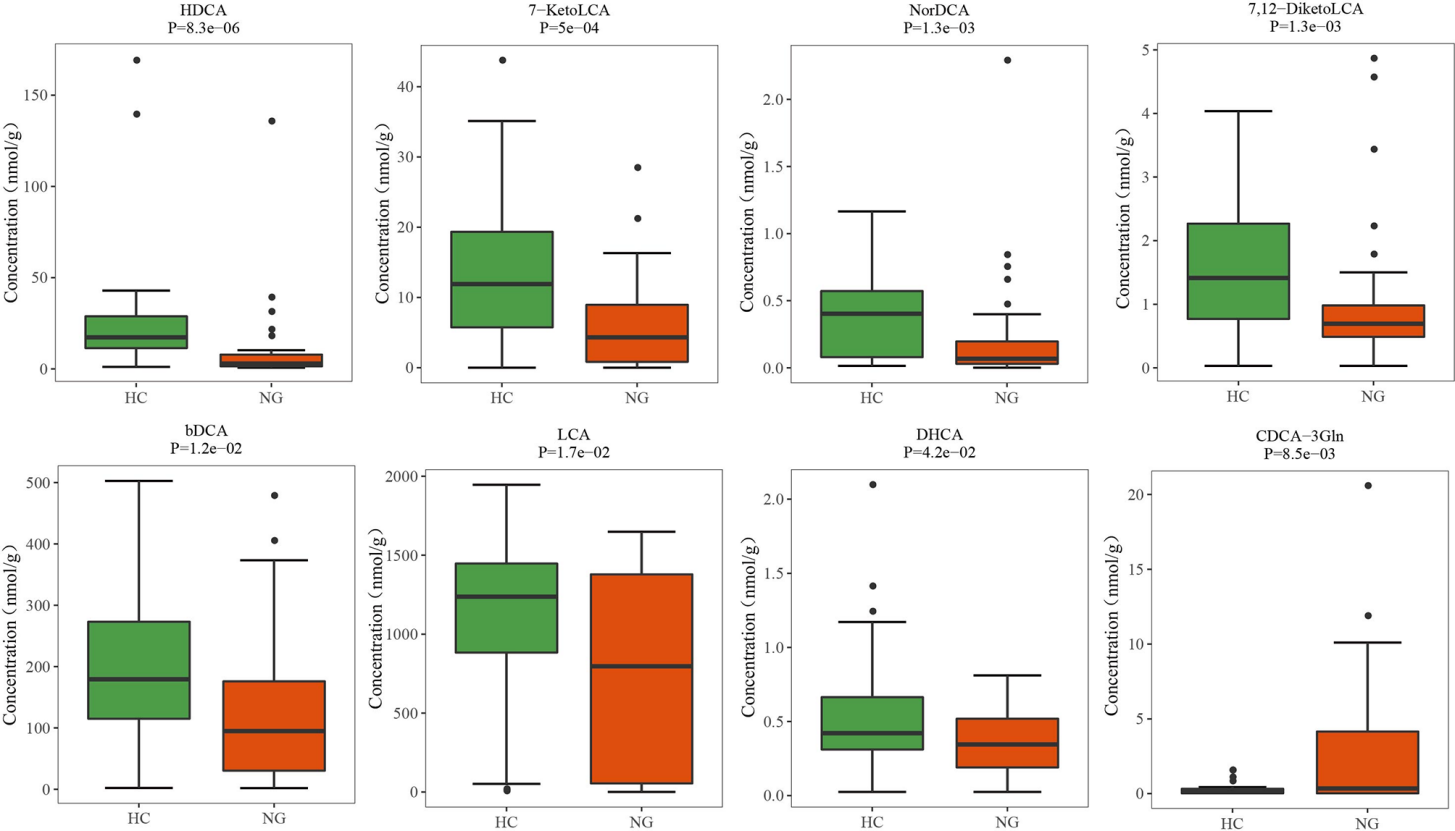
Altered bile acid profiles in the fecal samples of pediatric NAFLD patients. The quantitative concentrations of primary and secondary bile acids in the cohort of children with NAFLD (NG) and the healthy controls (HC) were determined by ultra-performance liquid chromatography coupled to tandem mass spectrometry (UPLC-MS/MS). Significant differences were measured by the Wilcoxon test. HDCA, α-hyodeoxycholic acid; 7-KetoLCA, 7-ketolithocholic acid; NorDCA, 23-nordeoxycholic acid; 7,12-DiketoLCA, 7,12-diketolithocholic acid; bDCA, 3-epideoxycholic acid; LCA, lithocholic acid; DHCA, dehydrocholic acid; CDCA-3Gln, chenodeoxycholic acid, 3-β-D-glucuronide.

The fecal bile acid profiles of 68 participants in this study were evaluated using a targeted metabolomics approach. The percentage of fecal secondary bile acids in NG children (57.16%) was significantly lower (P< 0.05) than that in HC children (74.40%; **Figure 4B**). The levels of all primary bile acids were elevated in NG children, although these increases were not statistically significant (**Figure 4A**). Specifically, the abundances of α-hyodeoxycholic acid(P< 0.001), 7-ketolithocholic acid (7-KetoLCA) (P< 0.001), 23-nordeoxycholic acid(P< 0.01), 7,12-diketolithocholic acid (7,12-DiketoLCA) (P< 0.01), 3-epideoxycholic acid (bDCA) (P< 0.05), LCA(P< 0.05), and dehydrocholic acid (DHCA) (P< 0.05) were significantly decreased in the NAFLD group compared to HC (**Figure 5**), with the exception of chenodeoxycholic acid-3-β-D-glucuronide (CDCA-3Gln), which was increased in NAFLD group. We also calculated two ratios that reflect enzymatic activities in the gut microbiota and liver, as previously reported, to better assess the enzymatic processes of bile acid metabolism that might underlie the differences found in children with NAFLD (MahmoudianDehkordi et al., 2019). Here, the CA/CDCA ratio (P=0.014) was found to be significantly higher in children with NAFLD, indicating that there was no shift in bile acid synthesis from the primary to the alternative bile acid pathway in the livers of children with NAFLD. While the ratio of DCA/CA was not significantly decreased in children with NAFLD, the LCA/CDCA ratio was significantly decreased (P=0.017), indicating a reduced production of secondary bile acids in these children. Ratios between primary and secondary conjugated bile acids showed similar trends, including TDCA/CA (P>0.05), GDCA/CA (P>0.05), GLCA/CDCA (P>0.05), and TLCA/CDCA (P=0.014) (**Table 2**).

**Table 2:** Ratios of bile acids reflecting the enzymatic activities in the gut microbiota and liver.

Metabolic process	Ratio calculated	Mean (95% CI)		P value (T test)
		HC (n=36)	NG (n=32)	
**Bile acid synthesis: primary *versus* alternative pathway**				
	CA/CDCA	1.550 (1.059, 2.042)	2.152 (1.477, 2.826)	**0.014**
	DCA/CA	108.698 (54.920, 162.476)	67.817 (17.624, 118.010)	0.168
	GDCA/CA	0.224 (0.121, 0.327)	0.164 (0.059, 0.269)	0.597
**Conversion from primary to secondary bile acid by intestinal bacteria**				
	TDCA/CA	0.092 (0.013, 0.171)	0.071 (0.001, 0,141)	0.647
	LCA/CDCA	64.639 (39.385, 89.893)	31.504 (9.266, 53.742)	**0.017**
	GLCA/CDCA	0.106 (0.026, 0.185)	0.050 (0.014, 0.087)	0.061
	TLCA/CDCA	0.042 (0.001, 0,084)	0.006 (0.002, 0.011)	**0.014**

CA, cholic acid; DCA, deoxycholic acid; GDCA, glycodeoxycholic acid; TDCA, taurodeoxycholic acid; CDCA, chenodeoxycholic acid; LCA, lithocholic acid; GLCA, glycolithocholic acid; TLCA, taurolithocholic acid; HC, healthy control; NG, NAFLD group; CI, confidence interval. A bold P value was considered significant.

### Disease-Associated Gut Microbiota Is Associated With Reduced Level of Fecal LCA in Children With NAFLD

**Figure 6:**
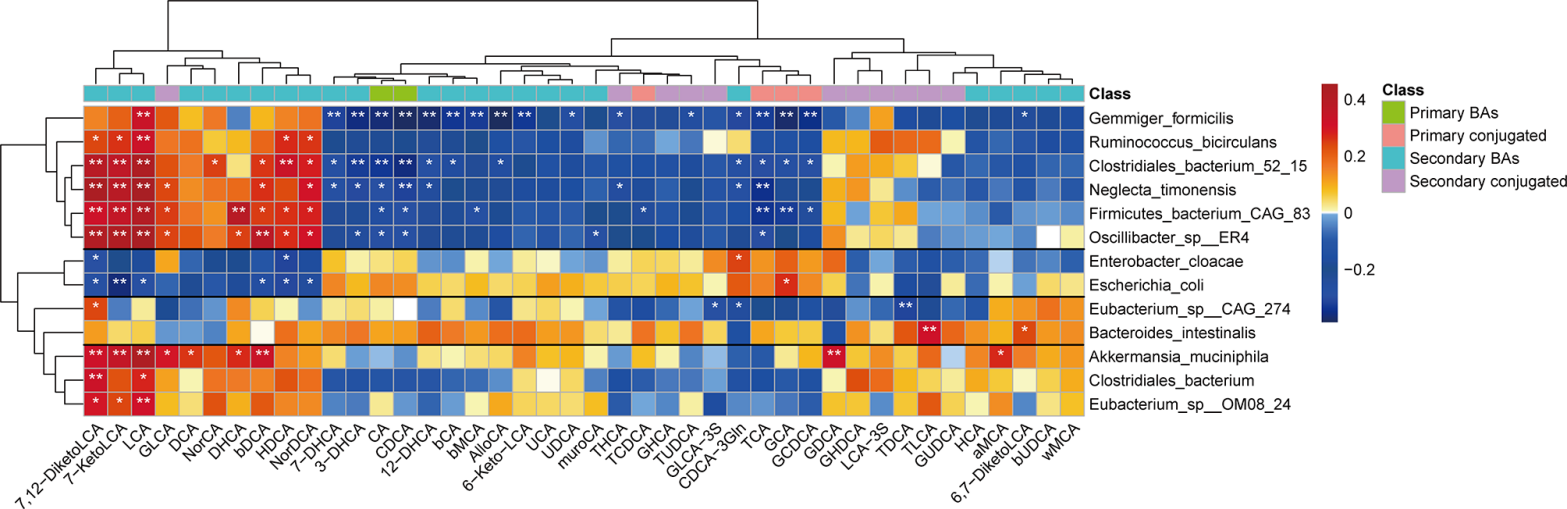
Spearman’s correlation analysis of fecal bile acids and the top 13 bacterial taxa at the species level with significantly distinct relative abundances between the two groups. Four different groups of bacteria taxa (groups G1 to G4) were observed. Blue and red represent the negative and positive correlations, respectively. *P < 0.05; **P < 0.01. MCA, muricholic acid; LCA, lithocholic acid; GLCA, glycolithocholic acid; TLCA, taurolithocholic acid; GCDCA, glycochenodeoxycholic acid; TCDCA, taurochenodeoxycholic acid; TCA, taurocholic acid; GCA, glycocholic acid; TDCA, taurodeoxycholic acid; GDCA, glycodeoxycholic acid; TUDCA, tauroursodeoxycholic acid; GUDCA, glycoursodeoxycholic acid; GHCA, glycohyocholic acid; THCA, taurohyocholicacid; GHDCA, glycohyodeoxycholic acid; DHCA, dehydrocholic acid; HDCA, hyodeoxycholic acid; GLCA-3S, glycolithocholic acid-3-sulfate acid.

To further determine the relationship between the gut microbiota and bile acids, we used Spearman’s rank correlation to compare the top 13 bacterial taxa at the species-level (those that were significantly changed in children with NAFLD) and fecal bile acids. We found that correlations between bacterial taxa and fecal bile acids clustered into four distinct groups (unsupervised groups G1 to G4; **Figure 6**). Species in Group G1, which included *G. formicilis*, *Ruminococcus bicirculans*, *Clostridiales bacterium 52_15*, *Neglecta timonensis*, *Firmicutes bacterium CAG:83*, and *Oscillibacter* sp. *ER4*, were positively associated with the levels of fecal LCA, 7-KetoLCA, and 7,12-DiketoLCA, and negatively correlated with fecal CA, CDCA, and conjugated primary bile acids. In contrast, species in Group G2, which includes *E. coli* and *E. cloacae*, were negatively associated with the levels of fecal LCA, 7-KetoLCA, and 7,12-DiketoLCA. Moreover, species in Group 4, which includes *A. muciniphila*, *C. bacterium*, and *Eubacterium* sp. *OM08_24*, were positively associated with the levels of fecal LCA, 7-KetoLCA, and 7,12-DiketoLCA. Overall, correlation analysis revealed that the abundance of *Eubacterium* sp. *OM08_24* (r=0.31; P<0.01) and *R. bicirculans* (r=0.32; P<0.01), which express bile acid-biotransforming-related genes, were positively correlated to the concentration of fecal secondary bile acid LCA, and the abundance of *E. coli* (r=-0.29; P<0.05) was negatively correlated to the concentration of LCA.

## Discussion

NAFLD is a disease that is associated with changes in gut microbial composition, function, and serum bile acid composition that are noteworthy in the understanding of NAFLD pathogenesis (Jiao et al., 2018; Zhao et al., 2019). In the present study, we applied a metagenomic sequencing approach and a UPLC-MS/MS system to pediatric NAFLD and HC subjects. We detected gut microbiota dysbiosis in the feces of children with NAFLD, which was characterized by an increase in the pathogenic species *E. coli*, *E. cloacae*, and *K. pneumoniae*, a decrease in potentially beneficial gut microbiota (*A. muciniphila* and *A. putredinis*), and lower abundances of the genera *Bacteroides*, *Eubacterium* and *Ruminococcaceae*, which are involved in bile acid metabolism (Song et al., 2019). In addition, we observed a decrease in fecal secondary bile acids in children with NAFLD compared to the HC group, which was strongly correlated with decreased abundance in *Eubacterium* and *Ruminococcaceae*. In our study, we observed an over-representation of potential pathogens such as *E. coli*, *K. pneumoniae*, and *E. cloacae*. Here, *E. coli* (LDA=4.37; P<0.001) showed the most marked increase in children with NAFLD. *E. coli* has previously been shown to confer potentially pathogenic effects upon their host, and its high abundance in patients’ guts indicates increased liver localization of LPS (Carpino et al., 2020). These potentially pathogenic species (*E. coli*, *K. pneumoniae*, and *E. cloacae*), with the capacity to produce endotoxins, may increase hepatic injury by inducing macrophage activation *via* the LPS-Toll-like receptor 4 (TLR4) pathway (Kapil et al., 2016; Carpino et al., 2020; Fei et al., 2020). In addition, *E. coli* and *K. pneumoniae* can also produce endogenous alcohol in the gut, which is known to increase gut barrier permeability, eventually resulting in liver damage (Zhu et al., 2013; Yuan et al., 2019). In contrast, compared to the healthy children, the species *A. muciniphila* (LDA=3.68; P<0.01) showed the most marked decrease in children with NAFLD. *A. muciniphila*, like *A. putredinis* (Ke et al., 2019), is thought to be involved in maintaining gut microbiota homeostasis and remodeling the diversity of gut microbiota through the production of short-chain fatty acids (SCFAs), such as propionic acid and acetic acid (van Passel et al., 2011; Kim et al., 2020). Additionally, *A. muciniphila* acts as a protective bacterium with the capacity to improve liver lipid synthesis and inflammation by repressing SREBP and IL-6 expression, respectively (Kim et al., 2020). It is thus believed that *A. muciniphila* and *A. putredinis* are in a group of beneficial gut bacteria. Decrease in *A. muciniphila* and *A. putredinis* is, therefore, an important indication of gut dysbiosis in NAFLD children.

Bile acid biotransformation by gut bacteria has received relatively little investigative attention in pediatric NAFLD. The first stage of bile acid metabolism in the small intestine, bile acid deconjugation, is catalyzed by gut bacteria with BSH activity. BSH deconjugates the taurine and glycine groups from conjugated bile acids, and subsequently reforms the primary bile acids CA and CDCA (Begley et al., 2005). The secondary enzymatic steps include 7α-dehydroxylation, which converts primary bile acids to secondary bile acids, such as DCA and LCA (Ridlon et al., 2006). BSH activity is widespread throughout most commensal bacteria inhabiting the intestine, and mainly within genera such as *Bacteroides, Lactobacillus, Eubacterium, Parabacteroides, Bifidobacterium*, and *Fusobacterium* (Song et al., 2019). In our study, we found that the abundance of BSH-active genera *Bacteroides* (*B. uniformis, B. fragilis, B. coprocola, B. intestinalis, B. thetaiotaomicron*, and *Bacteroides* sp. *D20*) and *Eubacterium* (*E. ventriosum, E. eligens*, and *Eubacterium* sp. *OM08_24*) were significantly decreased in children with NAFLD compared to HC, and the predicted KEGG data for gene abundance of BSH were consistent with the data of bacterial abundance described above. This indicates the gut microbiota alterations in children with NAFLD may affect the deconjugation processes of bile acid metabolism.

In addition, the known 7α-dehydroxylase-active bacteria, including *Eubacterium, Lachnospiraceae, Ruminococcaceae, Peptostreptococcus, Clostridiaceae*, and *Lactobacillus* (Pi et al., 2020), only account for a small portion of the intestinal tract of humans. However, these low-abundance gut bacteria may play a crucial role in increasing secondary bile acids in the human intestinal tract (Ridlon et al., 2016). In our study, we also found that the relative abundance of 7α-dehydroxylase-active gut bacteria, genus *Eubacterium* and *Ruminococcaceae*, were significantly reduced in children with NAFLD, although the detection of 7α-dehydroxylase baiCD gene abundance was not able to predict the very low levels of *Eubacterium* and *Ruminococcaceae*. The bile acid profiles showed a marked reduction in the levels of secondary bile acids (e.g., LCA) and a decrease in the ratio of secondary to primary bile acids (LCA/CDCA) in fecal samples from children with NAFLD. These data support the findings of bile acid-biotransforming-related gene abundance of gut microbiota.

Overall, the reduced secondary bile acids and related decreased gene expression demonstrated altered bile acid biotransformation in the *Bacteroides-, Eubacterium-* and *Ruminococcaceae-*depleted microbiota of children with NAFLD. Furthermore, these results might indicate elevated serum concentrations of bile acids or the presence of intrahepatic cholestasis, although the concentrations of serum total bile acid from clinical data showed no statistical difference between the two groups. However, our assumption might be in accordance with previously reported elevated serum concentrations of secondary bile acids in patients with NAFLD (Jiao et al., 2018). Interestingly, in our study, we also found that the predicted gene abundance for secondary bile acid biosynthesis (KEGG orthology ko00121) was higher in children with NAFLD than in healthy children, which may be related to the increased level of the secondary bile acid CDCA-3Gln. Correlation analysis revealed that the concentrations of secondary bile acid LCA were positively correlated with the abundance of the species *Eubacterium* sp. *OM08_24* and *R. bicirculans*, but negatively correlated with the potentially pathogenic *E. coli*. These data suggest that *Eubacterium-* and *Ruminococcus-*depleted gut microbiota along with imbalanced bile acid-transforming activity in children with NAFLD seemed more likely to influence the level of fecal LCA. Decreased abundance of the fecal *Eubacterium* and *Ruminococcus* genus in children with NAFLD indicated lower deconjugating activity and 7α-dehydroxylating activity, eventually contributing to the reduced level of LCA. Furthermore, Nascimento et al. revealed that LCA exhibits significant antibacterial activity, including inhibition of *E. coli* growth (do Nascimento et al., 2015). Our results of a reduced level of fecal LCA along with an enriched *E. coli* in the gut also implied a possible lower inhibition of LCA derived from the *Eubacterium-* and *Ruminococcus-*depleted microbiota on *E. coli* in the intestine, however, further investigations are necessary.

In addition, bile acid is known to act as a potent activator of transmembrane G protein-coupled receptor 5 [TGR5, also known as G protein-coupled bile acid receptor 1 (GPBAR1)]. TGR5 is a cell- surface receptor that is most strongly activated by LCA (Li et al., 2013). Our data showed that the level of LCA was significantly decreased in children with NAFLD, and this decrease could contribute to the inhibition of intestinal TGR5 signaling. TGR5 is widely expressed in human tissues, including the intestine, and is involved in lipid, glucose, and bile acid metabolism (Duboc et al., 2014). Finn et al. reported that intestinal TGR5 activation by specific TGR5 agonists could improve liver steatosis and insulin resistance in western diet-fed obese mice. In the intestine, activation of TGR5 can stimulate glucagon-like peptide (GLP) 1 (GLP-1) and 2 (GLP-2) secretion from enteroendocrine L-cells (Finn et al., 2019). Studies in American lifestyle-induced obesity syndrome (ALIOS) mouse models have shown that GLP-1 analog liraglutide treatment could improve insulin sensitivity and reduce hepatic lipid accumulation (Mells et al., 2012). In another mouse model of NASH, GLP-1 receptor agonist therapy could reduce liver steatosis and liver inflammation by reducing the influx of macrophages to the liver (Huang et al., 2010; Trevaskis et al., 2012). GLP-2 has also been shown to enhance intestinal epithelial barrier function, affecting both paracellular and transcellular permeation pathways, which may reduce local inflammation and improve NAFLD (Benjamin et al., 2000; Cani et al., 2009). Interestingly, Donepudi et al. (Donepudi et al., 2017) showed that TGR5-KO mice were protected from fasting-induced liver steatosis by decreasing liver fatty acid uptake and increasing liver fatty acid β-oxidation, likely through a novel pathway involving activation of the hepatic growth hormone-signal transducer and activator of transcription 5 (GH-Stat5) signaling. Taken together, the data imply an impaired intestinal bile acid–TGR5 signaling pathway in children with NAFLD and that gut microbiota dysbiosis orchestrates this abnormality. The low concentration of LCA in children with NAFLD may have clinical applications as a marker of pediatric NAFLD; however, further studies are required explore this concept.

Nevertheless, some study limitations in the experimental design need to be considered. First, the number of children enrolled in this study was relatively small, and it was also difficult to enroll children with similar dietary intakes, which may affect the composition of the gut microbiota and bile acid profile. Second, the current study did not investigate the serum bile acid profile and farnesoid X receptor (FXR) signaling. Both bile acid composition and FXR signaling in the serum could provide an accurate understanding of host bile acid metabolism and signaling. Third, investigating the effects of an *Eubacterium-* and *Ruminococcus-*depleted microbiota on intestinal bile acid metabolism in mouse models is a future direction for better understanding the pathophysiology of NAFLD.

In summary, the present study demonstrated a significant dysbiosis of the gut microbiota in children with NAFLD. We hypothesized that the disease-associated gut microbiota of children with NAFLD may contribute to the reduced the fecal concentration of secondary bile acids by affecting the enzymes that convert primary to secondary bile acids. These changes in the bile acid profile might be linked to impaired intestinal bile acid–TGR5 signaling, and these changes are associated with the pathogenesis of pediatric NAFLD. As this is a cross-sectional study and cannot demonstrate causality, our hypothesis should be investigated in further follow-up experimental studies. Integrating the gut microbiome together with metabolic features might provide a deeper understanding of gut microbiota-related conditions, from pathophysiological, therapeutic, and non-invasive diagnostic aspects, not only for pediatric NAFLD, but also for all gut microbiota- associated disorders.

## Data Availability Statement 

The datasets presented in this study can be found in online repositories. The names of the repository/repositories and accession number(s) can be found below: https://www.ncbi.nlm.nih.gov/, PRJNA730641.

## Ethics Statement 

The studies involving human participants were reviewed and approved by the Ethical Review Boards of the Second Affiliated Hospital and Yuying Children’s Hospital of Wenzhou Medical University. Written informed consent to participate in this study was provided by the participants’ legal guardian/next of kin.

## Author Contributions

JY, HZ, LC, YC, and QL designed the study. JY, HZ, LC, YR, and QL performed the experiments. JY, HZ, LC, YR, YC, and QL analyzed and interpreted the data. JY and QL wrote the first draft of the manuscript, and YC and QL revised the manuscript. All authors contributed to the article and approved the submitted version.

## Funding

This work was supported by the Research Fund for Lin He Academician New Medicine (18331212).

## Conflict of Interest

The authors declare that the research was conducted in the absence of any commercial or financial relationships that could be construed as a potential conflict of interest.

## Publisher’s Note

All claims expressed in this article are solely those of the authors and do not necessarily represent those of their affiliated organizations, or those of the publisher, the editors and the reviewers. Any product that may be evaluated in this article, or claim that may be made by its manufacturer, is not guaranteed or endorsed by the publisher.
